# Test–Retest Reliability of a Social Interaction Task

**DOI:** 10.3390/bs8100097

**Published:** 2018-10-22

**Authors:** Ekaterina Merkulova, Alexander Savostyanov, Andrey Bocharov, Ekaterina Proshina, Gennady Knyazev

**Affiliations:** 1 Laboratory of Psychophysiology of Individual Differences, Institute of Physiology and Basic Medicine, Novosibirsk 630090, Russia; a.n.savostyanov@physiol.ru (A.S.); bocharov@physiol.ru (A.B.); proshinaea@physiol.ru (E.P.); knyazev@physiol.ru (G.K.); 2 Humanitarian Institute, Novosibirsk State University, Novosibirsk 630090, Russia; 3 Laboratory of Psychological Genetics, Institute of Cytology and Genetics of SBRAS, Novosibirsk 630090, Russia

**Keywords:** social interactions, personality, test–retest reliability

## Abstract

Accurate repeatability of experimental data is the basis of professional scientific research. In this study we analyzed three consecutive experiments: The subjects had to complete a questionnaire three times under similar conditions within a 2–3 week interval to ensure reproducibility of the original data from experiment to experiment, using the method of test–retest reliability. Absolute reliability was assessed by the standard error of measurement (SEM) and smallest real difference (SRD). The relative reliability was estimated by calculating the intraclass correlation coefficients (ICC3,1) (average measures) and the results demonstrate almost perfect agreement. The social interaction model was applied for organization of the experimental study. In this virtual model, the participants had to choose one of three types of reactions (i.e., attacking, avoiding, or friendly) to stimuli-facial expressions (i.e., angry, fearful, sad, neutral, and happy). The results show significant correlation between personal characteristics and social interactions. The results of the influence of such personal characteristics as agreeableness, collectivism, extraversion, neuroticism, and those shown on the Relational-interdependent Self-Construal Scale and State-Trait Anxiety Inventory are highly consistent with other researchers’ data and common sense.

## 1. Introduction

Since human beings are known as “social animals”, social interactions are extremely important for a successful life. Cultural psychology and social neuroscience are constantly developing as independent disciplines, but it is important to understand the fact that various types of social statuses influence social interaction [1].

We all grow up, develop, and reveal ourselves in society. In connection with this, the investigation of human interactions is of great interest. The main limitation is the unexpected nature of human interaction. In this light, we need a well-controlled stimulus that makes it possible to minimize this difficulty in the experimental procedure. This work presents a model of social interaction. Hopefully, this model will assist in the investigation of behavioral reactions during virtual social interactions.

Social interaction depends on signals in different modalities. However, it is well known that facial expressions play a critical part in social interactions [2,3,4,5] because they are connected with emotions, a considerable factor in the decision making process, which is important before choosing a response [6,7]. Facial expressions of an emotion convey not only the internal state of the subject, but also convey interpersonal information, which is the predictor of social interaction [8]. The possibility of recognizing the feelings and attitude of other people is a critical factor in successful human interactions. The absence of this skill could lead to inappropriate social behavior.

To study the connections between individual characteristics and subject responses we used the Five Factor Model (FFM) of personality [9,10,11]. The Five Factor Model (FFM, Big Five personal characteristics) of personality includes five traits taken from the factor-analytic studies based on people’s descriptions of themselves and other people [12]. The FFM consists of five qualities: (1) Extraversion, defined as kind, sympathetic, friendly, and warm; (2) Conscientiousness, defined as dependable, reliable, thorough, and careful; (3) Openness to Experience, defined as artistic, creative, imaginative, and cultured; (4) Agreeableness, defined as friendliness, ability to come to an agreement, compassionate, and cooperative; and (5) Neuroticism, defined as anger, anxiety, depression, and vulnerability, with the opposite pole being emotional stability [13,14].

The question why different people manifest different kinds of behavior is the core problem of theories of personality [15]. Eysenck, Wilson, and Jackson [16] assumed that there are only three strategies in solving interpersonal problems. These three strategies are the following: Aggression and hostility, fear and flight, and social interaction. They suggested that these types of behavior are reflected in personality dimensions of psychoticism, neuroticism, and extraversion, respectively [17,18,19].

J.A. Gray’s theory [20,21,22] has two dimensions of personality; namely, anxiety and impulsivity. These personality features formed the basis for the conception of the Reinforcement Sensitivity Theory [23]. It contains two neurological systems, which are responsible for the reaction to environmental stimuli.

The behavioral inhibition system (BIS) is linked with an aversive motivational system. It is sensitive to punishment stimuli and responsible for the inhibition of behavior in risky situations. BIS is proposed to be a psychophysiological foundation (basis) of anxiety. The behavioral award system (BAS) is sensitive to reward stimuli and regulates reward-seeking behavior. BAS is assumed to be the basis of impulsivity [23]. The excessive activity of these systems is connected with psychopathological manifestations [24].

BIS is responsible for passive avoidance and has a weak role in active avoidance [25]. Gray considered that BIS is responsible for the experience of negative feelings such as fear, anxiety, frustration, and sadness in response to cues [26,27,28], and BAS is responsible for the experience of positive feelings such as hope, elation, and happiness [27,28].

A.J. Elliot and T.M. Thrash [29] were exploring the relationship between such important building blocks of personality as extraversion, neuroticism, BAS, and BIS, and came to the conclusion that these different features possess a common foundation of an approach and avoidance temperament. Approach and avoidance is a pair of concepts which refers to two basic orientations toward stressful information, or two basic modes of coping with stress [30].

Based on the above data we developed an experimental model, which is simplified to some extent, but on the other hand is a well-controlled task. In this study, we used a modified experimental model of virtual social interactions previously published in the study of Knyazev et al. [31]. Photos with emotional facial expressions, which contain angry, fearful, sad, happy, and neutral faces, were presented to participants. There are three types of social behavior that were offered in response: Friendly, avoiding, or attacking. A number of psychological questionnaires were requested to be filled out.

It is very important for researchers, especially in the fields of medicine and neurophysiology, to orient themselves concerning the issues of data reproducibility quality control, since false conclusions based on inappropriate methods of analysis lead not only to a waste of time for testing false hypotheses, but also to make threats to people’s health in the future.

An important parameter for determining the quality of a medical instrument is agreement with a reference point. Various statistical methods have been used to test for agreement. Agreement signifies the accuracy of that particular instrument [32]. The study in Reference [33] reviewed almost all statistical methods used to assess agreement of medical instruments measuring the same continuous variable in the medical literature. Some of these methods have been shown to be inappropriate: The Bland–Altman method [34], the correlation coefficient [35], coefficient of determination [36], paired *t*-test [37], regression coefficient [38], and Gradient-Based Algorithms for On-Line Regression [39] are all questionable [33].

All details about the correlation coefficient and coefficient of determination are described in the following book [40].

For our study we have chosen to use the ICC method. The main disadvantage of this method, that it is influenced by the range of data, is not the case with our data, which is why we have chosen it. If the variance between subjects is high, the value of the ICC will certainly appear to be high, and in our case the variance between subjects is sufficient. The use of ICC in assessing agreement has been criticized by Bland and Altman, who assert that the ICC ignores ordering, and treat the ICC method as a random sample from a population of methods [41].

Accurate reproducibility of the original data-repeatability or test–retest reliability [42] is the basis of professional scientific research [43], and this is generally recognized by the scientific community. Quality verification of the reproducibility of the original data is a standard section in scientific publications related to the statistical processing of experiments, and it is usual that the number of experiments should be no less than three within a 2–3 week interval [44].

As an absolute, the standard error of measurement (SEM) and smallest real difference (SRD) are used, which is equivalent to what is known as the “reliable change index” in psychotherapy research [45].

In this study we aimed to test two hypotheses:

**Hypothesis** **1.** *The experimental scheme and the chosen time interval between the measurements provide data with a sufficient level of reliability.*

**Hypothesis** **2.** *The model of social interaction that is a result of personal characteristics from questionnaires makes it possible to measure the real life parameters of behavior, such as attacking, avoiding, or friendly behavior. In the first approximation we can suggest that extraverts, as people with a lot of positive emotions, should offer friendship more often; aggressive people should be connected with attacking reactions; and anxious people should prefer avoiding behavior.*

## 2. Materials and Methods

### 2.1. Participants

Data was collected in a sample of 39 Caucasian men and women (mean age = 26.9; SD = 7.5; 61.5% females) who participated in the study.

The sample consisted of healthy, right-handed volunteers with normal or corrected to normal vision. We only included participants with no history of neurological or psychiatric disorders in the study. Each participant signed an informed consent. Participation was rewarded with a sum equivalent to about 5% of the monthly living wage. The study was approved by the Ethics Committee of the Research Institute of Physiology and Fundamental Medicine and was performed in accordance with the Declaration of Helsinki (1964). Written informed consent was obtained from all the participants.

### 2.2. Design of the Experiment 

All participants had to visit the laboratory three times. The interval between the visits was from two to three weeks. The photos were presented to the subjects on a (17 cm × 17 cm) computer screen, which was placed at a distance of 120 cm.

**Figure 1:**
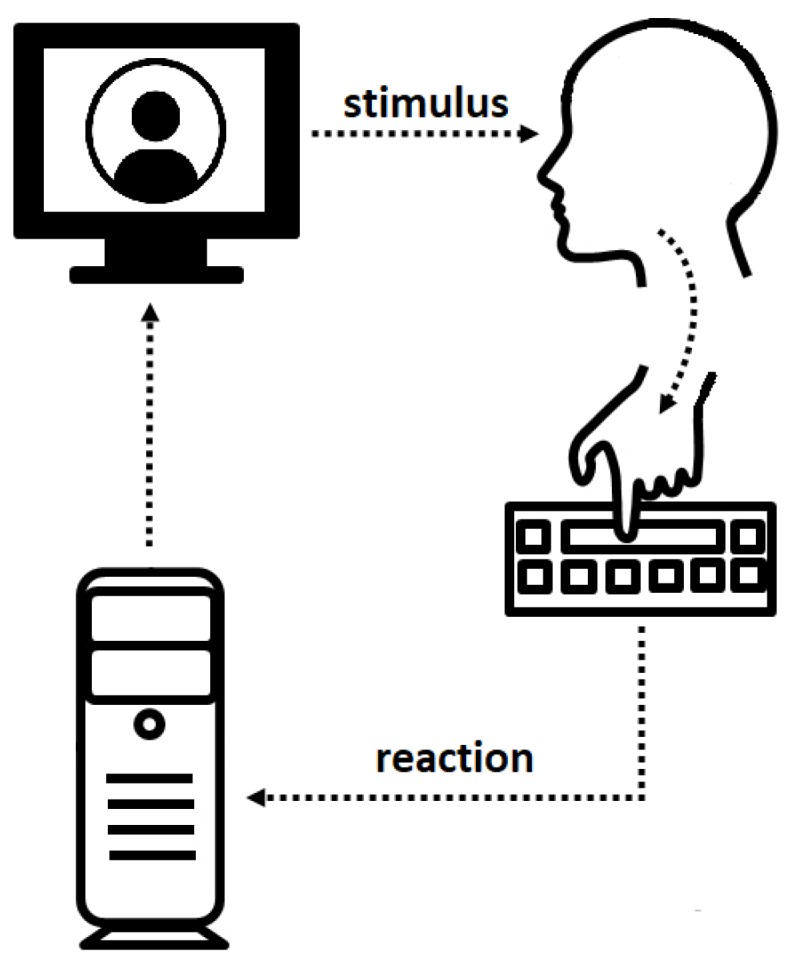
The experimental design.

**Figure 2:**
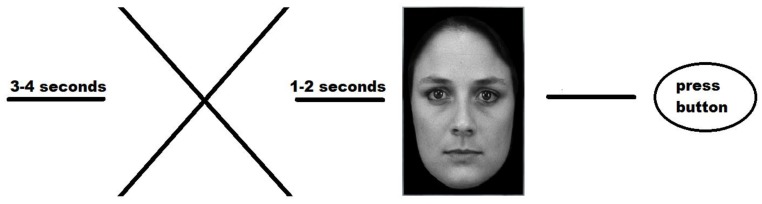
Diagram of one trial. After the fixation cross appeared for 1–2 seconds, the target stimulus (i.e., angry, fearful, sad, neutral, or happy face picture) was presented for about 2.5 seconds, until the subject chose one of the reactions (i.e., friendly, attacking, or avoiding).

The social interaction used a set of 200 black and white photographs of male and female faces as the stimuli. The photos were taken from the Karolinska Directed Emotional Faces database (KDEF, 2008). Our experiment included five types of emotions: Anger, happiness, sadness, fearful, and neutral. The photos were presented to the subjects (Figure 1) having been changed to black-and-white from color, with the faces presented against a black background. Original KDEF frontal view pictures were framed with an oval window to remove non-informative aspects (nonfacial areas) of the faces such as the hair and neck. We asked the participants to imagine that the faces which appeared on the screen were real people who they had to interact with (Figure 2). Three variants of reaction were possible for the subjects: (1) To offer friendship, (2) to attack, or (3) to avoid interaction. Each variant corresponded to the relevant button on the right side of the keyboard. The faces from different categories were presented in random order. One second before the presentation of a face, a cross as a ready signal appeared in the center of the screen. A prompt, listing the allowed variants of action, was presented at the bottom of the screen.

After the social interaction task, the subjects were asked to complete debriefed psychometric questionnaires. To measure personal characteristics we used the following questionnaires: The aggression questionnaire [46]; the validated Russian version of Goldberg’s “Big-Five factor markers” [47]; and the Self-Construal Scale (SCS) [48], which measured collectivism and individualism.

### 2.3. Statistical Analysis

#### 2.3.1. Analysis of Behavior and Effect of Personality Qualities

We used repeated-measures ANOVA with three within-subject factors: Visit (first, second, third), faces (happy, neutral, sad, fearful, aggressive), and choice (attacking, avoiding, friendly). These quantities were entered in the repeated measures ANOVA as factors to reveal effects of the within-subject factors. Personality variables were used as covariates. The Greenhouse–Geisser correction was used in order to avoid the risk of violating the sphericity assumption if necessary. Dependent variables were calculated in percentage terms for each of the three factor combinations.

We used a one-tailed test because we had two hypotheses, and this test for verification of significance was the most appropriate in this case.

For descriptive purposes, means and standard deviations (SD) were calculated for the variables of each visit.

#### 2.3.2. Behavioral Indicators

The variable in the model of test–retest reliability is considered to be the reaction coefficient. Five types of emotions expressed by facial expressions in the photos were used as items called ‘Stimuli’ in the model of test–retest reliability, and all deliveries of stimuli in each visit are the number Ntrials (Equation (1)). The attacking reaction coefficient is the number of attacking types of behavior divided by the Ntrials (Equation (2)), with the friendly (Equation (3)) and avoiding coefficients (Equation (4)) being calculated in the same way. The scale of reaction types is from attacking (–1) to avoiding (0) and friendly (1).

Nattacking + Nfriendly + Navoiding ≡ Ntrials,(1)

Rattacking = Nattacking/Ntrials,(2)

Rfriendly = Nfriendly/Ntrials,(3)

Ravoiding = Navoiding/Ntrials,(4)

#### 2.3.3. Analysis of Reliability 

We used test–retest reliability [33] to qualify reproducibility of the original data from experiment to experiment, and this method proved to be the most suitable for this in our case, and for our data with its own features. However, some researchers show that there are cases when such a check is made using inappropriate methods [33].

The relative reliability was estimated by calculating the intraclass correlation coefficients (ICC3,1) (average measures); the values in ICC3,1 between 0.81 and 1 demonstrate almost perfect agreement [49,50].

#### 2.3.4. Agreement

Absolute reliability was assessed by the standard error of measurement (SEM) and the smallest real difference (SRD). The formula of the SEM is:

(5) $$\mathrm{SEM}=\mathrm{SD}\times \sqrt{1-ICC}$$

Here SD is calculated taking into account all the subject’s data for each of three visits (Weir, 2005), and ICC is the ICC3,1. SRD was defined as the 95% confidence limit of the standard error of measurement (SEM) of the difference scores [51]:

(6) $$\mathrm{SRD}=\mathrm{SEM}\times 1.96\times \sqrt{2}$$

This value (index) is a measure of sensitivity to change, indicating the smallest within-person alteration in a score that can be considered to be a real change above any measurement error within one individual.

SPSS version 24.0 (IBM Corp., Armonk, New York, USA) was applied to calculate all the above statistics.

## 3. Results

### 3.1. Test–Retest Reliability

The ICC and agreement analyses were made based on a pattern at the end of the questionnaire. The subjects of the experiment had to go through this questionnaire three times in similar conditions within a 2–3 week interval.

The results of the experiment are represented in the tables below. The main information is in Table 1.

If the variables were the sum of all three types of reactions, and the stimuli were all five types of facial expression, we could see that only a friendly reaction to the different facial expressions showed bad results (mean = 0.499). This demonstrates that a friendly reaction to negative facial expressions (angry, afraid, sad) varies across the groups of subjects from visit to visit. Measures of response stability showed less variability between the test and retest for the avoiding reaction than for the angry and friendly reactions. In conclusion, all 3 visits demonstrated good test–retest reliability (mean = 0.741 in Table 1). However, greater differences would need to be observed between visits and friendly reactions to conclude that a real change occurred in measures obtained by social interactions (Table 2).

Repeated-measures ANOVA was used to analyze the effects of visits (3 levels), stimuli-faces (5 levels), and behavioral choice (3 levels) on the number of choices. There was no significant effect of the subject’s gender in this case.

**Figure 3:**
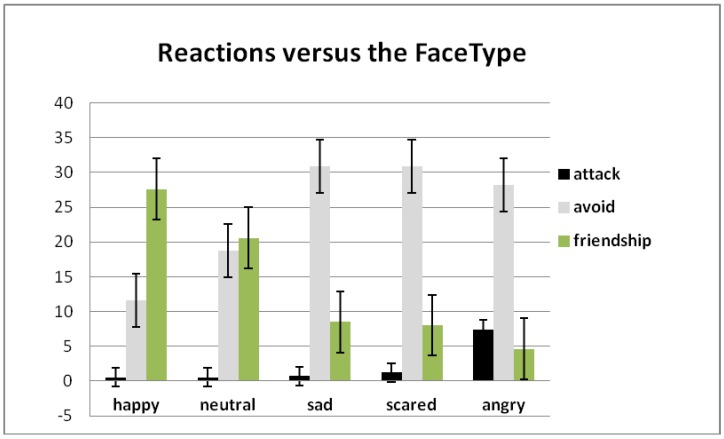
Influence of face type on choice (F (8, 296) = 41.185, *p* = 0.008, η2 = 0.077).

The main effect of choice (F (8, 296) = 57.186, *p* = 0.001, η2 = 0.601) showed that participants more frequently chose avoidance and friendship, and less frequently attack. Interaction analysis of choice × face (F (8, 296) = 50.342, *p* = 0.001, η2 = 0.570) showed (Figure 3) that participants more frequently attacked angry faces than others, offered friendship to happy faces, and more frequently avoided sad, fearful, and angry faces at the same time. The main effect visit × choice (F (4, 148) = 3.163, *p* = 0.016, η2 = 0.077) demonstrated that during the first visit people chose to attack more and to avoid less in comparison with other visits.

We are interested in the significant effect of personality throughout all three visits on the reaction choice, and different interactions within them. We therefore considered all personal variables in connection to the visits in terms of reactions and emotional types of presented faces.

The effects of extraversion and consciousness by the Big Five Method (BFM), the behavioral inhibition system in the Carver–White Questionnaire, and the individualism effect in the Self Comparison Scale in the questionnaire, were not significant.

**Figure 4:**
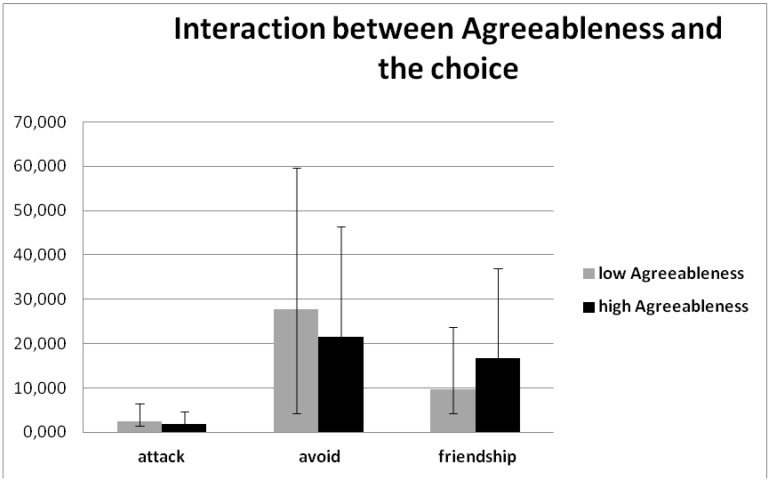
Interaction between the Agreeableness by the BFM and the choice (F (2, 74) = 5.507, *p* = 0.0005, η2 = 0.130).

A significant effect was found with Agreeableness by the BFM with choice, (F (2, 74) = 5.507, *p* = 0.019, η2 = 0.130), demonstrating the effect of less frequently avoiding and more often inviting to be friends, in the case of higher Agreeableness by the BFM (Figure 4).

**Figure 5:**
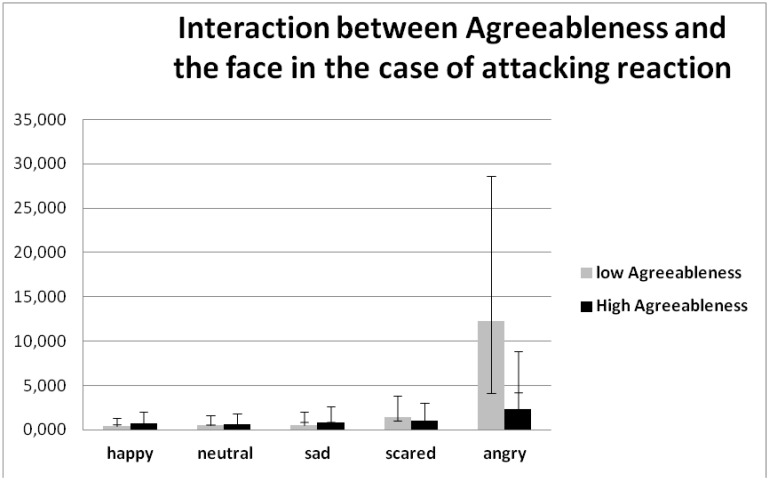
Interaction between the Agreeableness by the BFM with the choice and the face (F (8, 296) = 3.233, *p* = 0.002, η2 = 0.080).

The interaction between the Agreeableness by the BFM with the choice and the face (F (8, 296) = 2.559, *p* = 0.052 η2 = 0.065) reveals that people with high agreeableness tend to more often choose a friendly reaction to all types of faces, and choose avoiding reactions less frequently, except for aggressive faces (Table 3). They prefer to avoid aggressive people and attack less than the people with low Agreeableness. Almost the same rate of the attacking reaction to all types of faces was observed (Figure 5).

**Figure 6:**
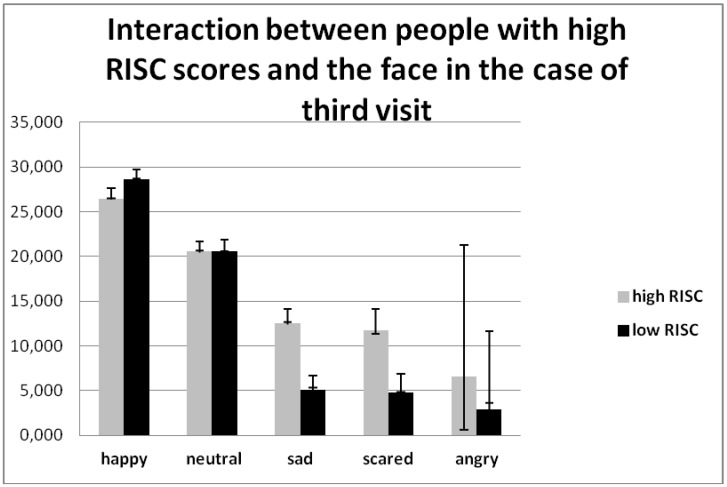
Interaction between people with high RISC scores and the face in the case of the third visit (F (8, 296) = 3.233, *p* = 0.001 η2 = 0.080).

There was also a significant interaction in the personal quality of relational-interdependent self-construal (RISC): Visit × face × RISC (F (8, 296) = 3.233, *p* = 0.002, η2 = 0.080). Although people with high RISC scores from the first visit to the third showed the main difference in their reaction type during the last visit, the first two visits were almost equal (Table 4). At the third visit they demonstrated an avoiding reaction more often (and there were more attacking reactions during the third visit for the group with low RISC), and less frequently offered to be friends to virtual persons (Figure 6).

**Figure 7:**
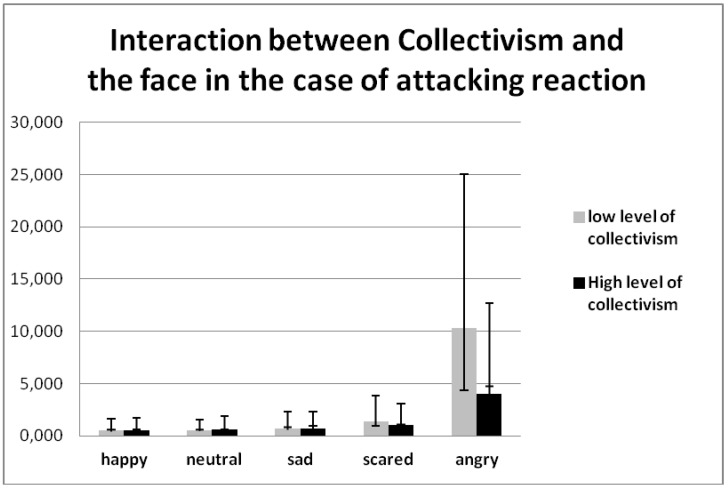
Interaction between collectivism, defined by the Self Comparison Scale choice × face (F (8, 296) = 3.233, *p* = 0.001 η2 = 0.080).

A significant effect with collectivism, defined by the Self Comparison Scale choice × face × collectivism (F (8, 296) = 3.233, *p* = 0.002, η2 = 0.080), demonstrates that people with a high collectivist level are more friendly to everybody, but they avoid happy and aggressive types of faces more in comparison with other facial types (Table 5). Additionally, people with a low collectivist level were more likely to demonstrate an attacking reaction to fearful and aggressive people as compared with highly collectivist people (Figure 7).

**Figure 8:**
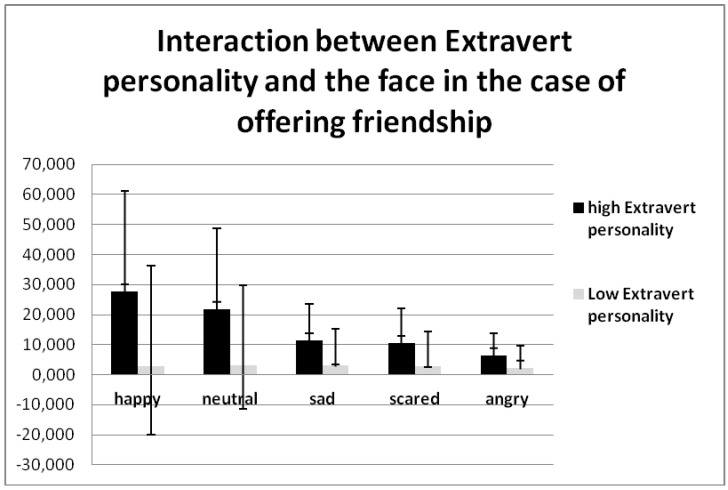
Interaction between the extraversion personality × face × choice (F (8, 272) = 0.949, *p* = 0.0545 η2 = 0.056).

A significant effect in extraversion by the Eysenck Personality Profiler × face × choice (F (8, 272) = 0.949, *p* = 0.0545 η2 = 0.056) was noted, demonstrating the fact that people with a high extraversive attitude in their life attack (Figure 8) and avoid (Table 6) almost all types of faces less often than people with a lower extravert personality. However, they offer friendship more often than the opposite group (Table 6).

**Figure 9:**
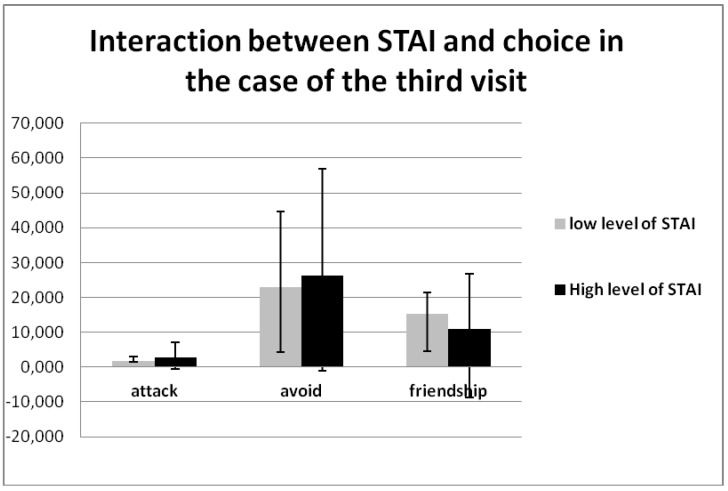
Interaction between the State-Trait Anxiety Inventory (STAI) × choice × visit (F (4, 148) = 2.886, *p* = 0.031, η2 = 0.078).

The personal quality of State-Trait Anxiety Inventory (STAI) × choice × visit (F (4, 148) = 2.886, *p* = 0.031, η2 = 0.078) was also researched, demonstrating a significant effect with visits and choice. This effect tells us that people with high STAI more often avoid others, and less frequently offer friendship from visit to visit (Table 7, Figure 9).

**Figure 10:**
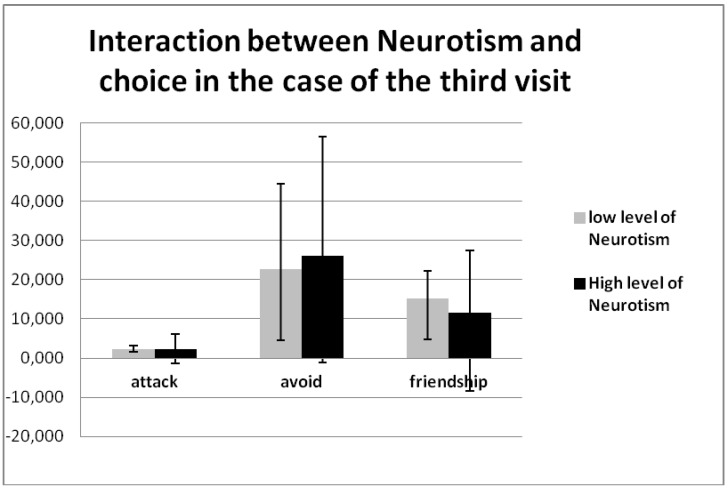
Interaction between neuroticism × choice × visit (F (4, 136) = 2.912, *p* = 0.012, η2 = 0.079).

The authors of this article also considered the neuroticism quality by Goldberg, and found a significant effect of neuroticism × choice × visit (F (4, 136) = 2.912, *p* = 0.012, η2 = 0.079). As with the previous effect with the STAI quality, this effect demonstrates that people with high neuroticism qualities more often avoid others, and less frequently offer friendship from visit to visit. We demonstrated this effect only on the third visit (Table 8, Figure 10).

## 4. Discussion

The main aim of this study was to define the connections between the personality parameters of people in tests and their reactions to facial expressions presented to them.

We considered the connection between the reaction of the subject generated by different facial expressions according to the theory of Eysenk, and have received significant results with such parameters as agreeableness, collectivism, emotional intelligence, RISC, extrovertism, STAI, anger, and neuroticism, and the reaction types (attacking, avoiding, and friendly), which is further proof that the individual characteristics of a person predict social behavior. These results are consistent with the extension of the neural efficiency concept for personality [45], and it complies with many works listed below. 

Another goal of this study was to check the test–retest reliability connected with repeatability of the experiments, and it was found that it is almost perfect. This means that the more reliable the received results are, the more probable the predictions are.

The personality quality of agreeableness makes people think that this quality is the key quality for proactive behavior, and the results of another study of this laboratory confirms this feature as well [52]. In this relation we can affirm that agreeableness is a personality factor which undoubtedly predicts interpersonal deviations. Agreeableness is related to friendly altruistic behavior and avoiding competition, anger, egocentrism, and jealousy [53,54,55,56], and it is connected with social-cognitive Theory of Mind [57].

Research on the “Big Five” personal characteristics and aggressive behavior has discovered that people with low agreeableness are more aggressive and violent [58]. In the results it can be seen that people with low agreeableness like to attack more frequently than people with high agreeableness, and they attack aggressive faces least of all.

Agreeableness is related to cooperativity [59,60], and our results demonstrate that people with high agreeableness have a greater desire to make friends, and less wish to attack and avoid other people. That is, they try to fit in with the collective and to organize groups and friendly interaction, which means cooperativity.

Overall, individuals unconsciously use impressions and clues to predict to which extent they can trust the interaction partner and to behave correspondingly [61,62,63]. Being naturally inclined to cooperation, agreeable individuals are willing and able to do that. It is suggested that the propensity to delve into the mental states of other people seems to be central to agreeableness [57]. Highly agreeable people obviously tend to make decisions based on emotions more quickly in contrast with less agreeable people [64].

At the beginning of this article we made a hypothesis about the extravert personality and our results confirmed it. Optimistic people have high extravertism in their personality [65] and a positive affect association [66]; that is why they offer friendship more often and avoid others less often than people who are the opposite.

We can also combine STAI and neuroticism, though they have the same effect. Our results with the quality of anger have the opposite effect in comparison with neuroticism. Anger is inversely related to neuroticism [67]. STAI and anger qualities are very important characteristics in the diagnosis of clinical disorders, and these parameters are connected with the emotional condition of individuals, which can be the reason for the reaction [68].

According to the article of Reference [69], anxiety and aggressiveness may predispose one to exaggerate hostile intentions in other people. This may have an impact on everyday interpersonal relationships, as our results demonstrate; that is, people with a high neurotic level avoid other people more often, and less often offer friendship.

Extraversion, neuroticism, and agreeableness predicted measures of friendship quality. Therefore, our results allow us to conclude that variations in affectivity are significant predictors of qualitative aspects of people’s social relationships [70].

The next personality trait, that of collectivism, is interesting to view and compare in different cultures. For example, in some studies [71] it can be seen that there is no definite differences between Japanese and USA cultures, but there are significant differences in the personal characteristics of individualistic-collectivistic groups in brain activities [72,73,74,75]. It is remarkable that in our investigations we have found significant differences between the collectivist trait and reaction, and moreover, people with a low level of the collectivist trait attack aggressive people more often. This theme of collectivism-individualism is also described using details of anatomic structure in fMRI research [76]. Incidentally, people with the collectivist trait need less time to transition to another state, and the dynamic of emotions is higher in such people than in the group of people with the opposite trait [77,78].

The results of this study demonstrate that people with high RISC qualities offer friendship more often, they are more communicative, and suggest being friendly with every type of face more than those low in RISC. They report more relationship supportive behaviors, experience greater fulfillment of friendship functions, and report higher relationship quality [79]. They are related to more positive evaluations of the relationship [80] than individuals low in RISC.

The last quality we would like to consider is the STAI. Our results are in accordance with the hypothesis that personal characteristics, such as anger, rather accurately describe the reaction of the person. The Five Factor personality test significantly predicts the trait of anger and anger expression styles (anger-in, anger-out, and anger control) [81]. Furthermore, people with high STAI avoid others and offer friendship less frequently, because they really do not know what to expect from others, and are probably afraid of others forming a wrong opinion of them, because such people have the wrong feedback [82].

## 5. Conclusions

In conclusion, this study shows that the connection between emotional social stimulus-facial expressions and behavioral response-reactions is very strong, which is confirmed by a high level of test–retest reliability.

This study has a number of limitations. First of all, the test–retest reliability has some misrepresentation in its number because of the mutilation social interaction model; that is, with the friendly reaction, almost nobody wants to become friends with an angry or fearful person, which is why there are a lot of zeros in the summed variables and a low coefficient of test–retest reliability. Second of all, we understand that virtual social interaction with presented facial expressions, which were developed with the help of the Karolinska Directed Emotional Faces database [83], is rather doubtful. Further, we hope to do experiments with real people (more close to social reality). 

## Figures and Tables

**Table 1:** Mean (± SD) values, mean signed difference (MSD), standard error of measurement (SEM), and smallest real difference (SRD) for each visit. The value of the variable acted as the reaction coefficient.

Stimulus	Friendly	Avoiding	Attacking
ICC3,1	0.687	0.825	0.713
Lower bound	0.531	0.722	0.565
Upper bound	0.810	0.899	0.828
Mean ± SD	0.35 ± 0.18	0.58 ± 0.02	0.05 ± 0.07
SEM	0.100	0.008	0.037
SRD	0.279	0.023	0.103

**Table 2:** The intraclass correlation coefficient for the three visits of the social interaction between all reaction and stimuli.

Stimulus	Friendly	Avoiding	Attacking
	**Angry**	**Angry**	**Angry**
ICC3,1	0.366	0.811	0.776
Lower bound	0.163	0.701	0.652
Upper bound	0.570	0.890	0.868
Mean ± SD	1.38 ± 0.26	0.88 ± 0.21	0.026 ± 0.004
SEM	0.207	0.091	0.002
SRD	0.574	0.253	0.005
	**Fearful**	**Fearful**	**Fearful**
ICC3,1	0.343	0.818	0.811
Lower bound	0.139	0.712	0.701
Upper bound	0.550	0.895	0.890
Mean ± SD	1.02 ± 0.36	1.14 ± 0.32	0.027 ± 0.004
SEM	0.292	0.137	0.002
SRD	0.809	0.378	0.005
	**Sad**	**Sad**	**Sad**
ICC3,1	0.394	0.781	0.830
Lower bound	0.192	0.659	0.729
Upper bound	0.594	0.872	0.902
Mean ± SD	0.42 ± 0.36	1.51 ± 0.32	0.063 ± 0.009
SEM	0.280	0.150	0.004
SRD	0.777	0.415	0.010
	**Neutral**	**Neutral**	**Neutral**
ICC3,1	0.564	0.758	0.799
Lower bound	0.379	0.619	0.684
Upper bound	0.725	0.857	0.883
Mean ± SD	0.40 ± 0.30	1.51 ± 0.14	0.006 ± 0.01
SEM	0.198	0.069	0.000
SRD	0.549	0.191	0.001
	**Happy**	**Happy**	**Happy**
ICC3,1	0.832	0.758	0.870
Lower bound	0.732	0.626	0.788
Upper bound	0.903	0.857	0.926
Mean ± SD	0.23 ± 0.21	1.32 ± 0.18	0.37 ± 0.26
SEM	0.086	0.089	0.094
SRD	0.239	0.245	0.260

**Table 3:** Interaction between agreeableness and the face in the case of attacking, avoiding, and friendship reactions.

Stimulus	Friendly	Avoiding	Attacking
	**Angry**	**Angry**	**Angry**
low agreeableness	1.000	26.900	12.250
high agreeableness	8.439	29.632	2.316
	**Fearful**	**Fearful**	**Fearful**
low agreeableness	3.250	35.617	1.433
high agreeableness	13.053	25.807	1.018
	**Sad**	**Sad**	**Sad**
low agreeableness	2.817	36.467	0.550
high agreeableness	14.491	24.912	0.860
	**Neutral**	**Neutral**	**Neutral**
low agreeableness	16.133	23.583	0.517
high agreeableness	25.298	13.579	0.596
	**Happy**	**Happy**	**Happy**
low agreeableness	24.867	14.233	0.383
high agreeableness	30.491	8.825	0.684

**Table 4:** Interaction between people with high and low RISC scores during all three visits.

Stimulus	First Visit	Second Visit	Third Visit
	**Angry**	**Angry**	**Angry**
low RISC	13.095	13.381	13.540
high RISC	13.852	13.519	13.185
	**Fearful**	**Fearful**	**Fearful**
low RISC	13.460	13.063	13.429
high RISC	13.556	13.315	13.389
	**Sad**	**Sad**	**Sad**
low RISC	13.270	13.349	13.413
high RISC	13.222	13.352	13.481
	**Neutral**	**Neutral**	**Neutral**
low RISC	13.730	13.476	13.175
high RISC	12.759	13.019	13.481
	**Happy**	**Happy**	**Happy**
low RISC	13.111	13.397	13.111
high RISC	13.278	13.463	13.130

**Table 5:** Interaction between collectivism and the face in the case of attacking, avoiding, and friendship reactions.

Stimulus	Friendly	Avoiding	Attacking
	**Angry**	**Angry**	**Angry**
low Collectivism	2.937	26.635	10.349
high Collectivism	6.593	30.093	3.981
	**Fearful**	**Fearful**	**Fearful**
low Collectivism	4.825	33.603	1.413
high Collectivism	11.759	27.611	1.019
	**Sad**	**Sad**	**Sad**
low Collectivism	5.095	34.524	0.730
high Collectivism	12.481	26.537	0.667
	**Neutral**	**Neutral**	**Neutral**
low Collectivism	20.587	19.095	0.492
high Collectivism	20.611	18.259	0.630
	**Happy**	**Happy**	**Happy**
low Collectivism	28.571	10.619	0.524
high Collectivism	26.481	12.741	0.537

**Table 6:** Interaction between extravert personality and the face in the case of attacking, avoiding, and friendship reactions.

Stimulus	Friendly	Avoiding	Attacking
	**Angry**	**Angry**	**Angry**
low Extravert personality	2.372	31.021	6.854
high Extravert personality	6.533	2.600	2.369
	**Fearful**	**Fearful**	**Fearful**
low Extravert personality	2.806	33.646	0.958
high Extravert personality	10.433	2.490	0.343
	**Sad**	**Sad**	**Sad**
low Extravert personality	3.087	33.271	0.583
high Extravert personality	11.300	2.710	0.282
	**Neutral**	**Neutral**	**Neutral**
low Extravert personality	3.063	18.896	0.521
high Extravert personality	21.917	2.719	0.186
	**Happy**	**Happy**	**Happy**
low Extravert personality	2.695	11.000	0.500
high Extravert personality	27.617	2.346	0.199

**Table 7:** Interaction between people with a high and low level of STAI and choice during all three visits.

Stimulus	First Visit	Second Visit	Third Visit
	**Attack**	**Attack**	**Attack**
low level of STAI	1.863	1.695	1.832
high level of STAI	2.129	1.706	2.788
	**Avoid**	**Avoid**	**Avoid**
low level of STAI	22.358	23.400	22.937
high level of STAI	22.247	25.882	26.212
	**Friendship**	**Friendship**	**Friendship**
low level of STAI	15.779	14.905	15.232
high level of STAI	15.624	12.412	11.000

**Table 8:** Interaction between people with a high and low level of neuroticism and choice during all three visits.

Stimulus	First Visit	Second Visit	Third Visit
	**Attack**	**Attack**	**Attack**
low level of Neuroticism	2.188	2.247	2.212
high level of Neuroticism	1.811	1.211	2.347
	**Avoid**	**Avoid**	**Avoid**
low level of Neuroticism	21.059	22.765	22.671
high level of Neuroticism	23.421	26.189	26.105
	**Friendship**	**Friendship**	**Friendship**
low level of Neuroticism	16.753	14.988	15.118
high level of Neuroticism	14.768	12.600	11.547
